# Editorial: Spatial and regional mapping of brain neural communication

**DOI:** 10.3389/fncel.2026.1880807

**Published:** 2026-06-03

**Authors:** Janet L. Paluh, Debayan Das, Amitava Mukherjee

**Affiliations:** 1 Nanoscale Science and Engineering, College of Nanotechnology, Science and Engineering, University at Albany, State University of New York, Albany, NY, United States; 2 Indian Institute of Science, Bangalore, India; 3 Birla Institute of Technology and Science - Pilani, Dubai Campus, Dubai, United Arab Emirates

**Keywords:** artificial intelligence, clinical imaging, machine learning, neurophysiology, neuroscience

## Background

Resolving the interconnectivity of the brain in line with its exquisite architecture and dynamic developmental and adaptive framework represents the next frontier in artificial neural networks and agentic artificial intelligence (AI) systems. Research that focuses on compartmentalized signaling modules and their spatial integration contributes to a hollistic model of brain neural networks from synapses to functional outcomes. As we begin to define spatial information processing and plasticity in a multicellular, multidomain anatomy, integrating across scales and data types is needed. By exploring the underlying nature of neural communication in focused studies of specialized subnetworks or in normal vs. perturbed-system cases, foundational frameworks and paradigms can be established to help guide next steps. Such studies will help to reveal the broader scope of brain function. This includes an astonishing adaptability to readjust signaling and navigate neurodevelopmental challenges or as an adult addiction or diseases, thereby informing on mechanisms accounting for redundancies, learning, adaptability, recovery, and limitations.

Neuroimaging tools track whole brain currents, oscillations, and synchrony of neural networks, for example identifying regional electrical activity directly via electroencephalography (EEG) or indirectly via associated magnetic fields (MEG) or hemodynamic response in functional magnetic resonance imaging (fMRI). Challenging in real time is to interlink global studies with microscale and molecular mechanics and transcriptomic foundations. This includes timescales of neural communication that are not uniform but may vary by region across distinct brain anatomy and be further nuanced by the specific perturbation. This complexity across scales drives the continual need for technical and model integration to fully reconstruct the intrinsic and task-evoked nature of brain neural networks.

By exploring mechanistic hypotheses with complementary modeling studies, advanced AI algorithms and hardware co-integration and even perhaps linking AI with quantum computing we can bridge gaps. Neuron modeling is perhaps most advanced and enables exploration of dendritic arbors for multisynaptic inputs to neural signaling. Less advanced is the ability to address plasticity systemically with multiple neurons networked into different regions with different inputs or to factor on a grand scale additional mechanisms for signal maturation, stabilization, removal, or reinforcement. Even beyond synaptic level questions are metabolic contributions and impacts occurring when the blood-brain-barrier is collapsed in injury or disease. Thus, while models may allow us to more quickly test theories on rebalancing and reweighting of neural signaling functions from both bottom up and a top down contributions, many challenges still remain in compiling a digital twin of the brain and neuronal connectivity.

Our Research Topic includes four articles that fit to our scope. These studies examined new human brain imaging fMRI data or public resources such as the Human Connectome to address brain neural networks related to functional outcomes and tested their hypotheses. Their studies also reexplore previously published studies in these topic areas, seeking to reconcile in some cases contrasting models on neural communication networks by refocused evaluation of the data and employing computational models to uncover new details. This exciting group of papers highlights the importance of not only neural network dynamics, but also how the field itself continues to make important strides beyond a blueprint of the brain anatomy to an evolving, learning, adapting, signaling framework to deliver functional precision.

Wu and Gollo focused on the multiscalar nature of intrinsic neural timescales (INT) of networked neurons in subcortical–cortical regions and the link to dendritic size as a biomarker for brain signaling states. fMRI studies on healthy young adults was done to define INT information processing rates and then correlate with dendritic arbor size. Digitized neurons (via Neuromorph) were analyzed with and without progressive dendritic arbor pruning typical of neurodegeneration. Excessive pruning was consistent with a loss of predicted neuronal firing rates and increased intrinsic timescales, presumably from lost opportunities for synaptic input and thus longer information processing.

Yao et al. looked at whole brain functional networks of treatment-naive blepharospasm patients and healthy controls by applying resting state fMRI and graph theoretical analysis. The study defined patterns of disruption to the network topology that included decreased use of regions and bidirectional connectivity changes in patients. Findings support a large scale network disorder of sensorimotor circuits and cortico-basal ganglia thalama-cortical loops.

Sun et al. explored neurological substrates for behavioral extraversion that include tendency toward assertiveness, positive emotionality, sociability and sensation seeking using retrospective analysis. Neuroimaging data was re-evaluated based on functional connectivity network mapping (FCNM) and fMRI data from the Human Connectome Project to generate a system-level perspective linked to nodes within the frontoparietal and default mode networks (FPN, DMN, respectively).

Wang et al. applied network mapping to understand heterogenous correlates of neuroticism that present as emotional instability and vulnerability and are often linked to the prefrontal cortex (PFC) and regional gray matter volume. By cross-modal associations between brain networks and neurotransmitter atlases their study highlights interconnected brain regions links to neuroticism. Key brain signaling networks include the DMN and Ventral attention network (VAN), highlighting the importance of VAN in neuroticism vulnerability, its potential as a therapeutic-target, and regional links to 5-HT2A, CBI and mGluR5.

These studies demonstrate and refine brain region networks with functional outcomes in behavioral neuroticism, extraversion and adult onset dystonia taking advantage of global and regional neuroimaging maps, neurotransmitter atlases and computational modeling. The next critical steps will require microscaling of that regional information to understand neuron-neuron communication as well as spatial transcriptomics to generate information on genotype-connectome outcomes. The dynamic basis of neural communication and plasticity coupled across scales will be facilitated by development of expanded data resources and their interconnectivity via computational models and AI to bring synergistic advances in linked to behavior, aging and disease.

